# Genomic Analysis Highlights the Misinterpretation of Acquired Aminoglycoside Resistance Genes in *Deinococcus radiodurans*

**DOI:** 10.3390/cimb48050505

**Published:** 2026-05-14

**Authors:** Gabriel Augusto Marques Rossi, Fábio Parra Sellera, Eliana Guedes Stehling, João Pedro Rueda Furlan

**Affiliations:** 1 Department of Veterinary Medicine, University Vila Velha (UVV), Av. Comissário José Danta de Melo, n.21, Vila Velha 29102-920, ES, Brazil; 2 School of Veterinary Medicine, Metropolitan University of Santos, Santos 11080-100, SP, Brazil; fsellera@usp.br; 3 Department of Internal Medicine, School of Veterinary Medicine and Animal Science, University of São Paulo, São Paulo 05508-270, SP, Brazil; 4 Department of Clinical Analyses, Toxicology and Food Science, School of Pharmaceutical Sciences of Ribeirão Preto, University of São Paulo, Av. do Café, S/N, Monte Alegre, Ribeirão Preto 14040-903, SP, Brazil; elianags@usp.br; 5 Department of Pharmaceutical Sciences, Health Sciences Center, Federal University of Paraíba, João Pessoa 58051-900, PB, Brazil

**Keywords:** antimicrobial resistance, whole-genome sequencing, resistome analysis, comparative genomics, vector integration, recombination capacity

## Abstract

Aminoglycoside resistance is commonly mediated by enzymatic modification, target alteration, or efflux mechanisms; however, acquired resistance has not been characterized in radiation-resistant *Deinococcus* species. Here, we investigated the occurrence and genomic context of acquired aminoglycoside resistance genes in all publicly available *Deinococcus radiodurans* genomes. A total of 19 genomes were screened using ResFinder and CARD, followed by comparative genomic analyses. The *aadA1* gene was identified in two genomes, being located on the plasmid pSP1 in strain R1 dM1, a known shuttle vector used for genetic manipulation. In contrast, *aadA1* was found on a chromosomal contig in strain DRR11, suggesting a possible assembly artifact. Additionally, the *aph(3′)-Ia* gene was detected in three genomes within a conserved chromosomal region that lacks this gene in reference strains. Sequence similarity analyses indicated that *aph(3′)-Ia* is associated with laboratory vectors, being consistent with a potential non-natural origin. Considering the high recombination capacity and genomic plasticity of *D. radiodurans*, these findings suggest that the detected aminoglycoside resistance genes may be derived from laboratory constructs, potentially combined with assembly inconsistencies or chromosomal integration events. Therefore, this study highlights the importance of integrating genomic context with molecular and evolutionary plausibility to avoid misinterpretation of antimicrobial resistance in extremophiles and model organisms, and underscores the importance of complementary raw-read analyses to distinguish natural acquisition from technical or laboratory-derived origins.

## 1. Introduction

*Deinococcus radiodurans* is an extremophilic bacterium widely recognized for its resistance to ultraviolet and ionizing radiation, desiccation, and oxidative stress. This remarkable resilience is largely attributed to highly efficient DNA repair systems, robust antioxidant defenses, and sophisticated cellular mechanisms that protect proteins and nucleic acids from oxidative damage [1,2]. Because of these properties, *D. radiodurans* has become a model organism for studies on stress tolerance and has attracted interest for biotechnological and environmental remediation applications, particularly in radiation-contaminated or other extreme environments [2,3].

Despite extensive studies on stress resistance mechanisms, little attention has been given to the antimicrobial susceptibility profile of *D. radiodurans* and its potential to acquire antimicrobial resistance (AMR). A previous investigation has shown that this species is generally susceptible to many classes of antimicrobials, although genomic analyses have identified genes potentially associated with AMR, such as multidrug efflux transporters and ABC transporter systems [4]. The natural competence and ability of *D. radiodurans* to acquire foreign DNA suggest that this species may act as a reservoir or recipient of resistance determinants under selective pressure [2]. Moreover, environmental bacteria are increasingly recognized as important reservoirs of AMR determinants and may contribute to the dissemination of AMR across ecological environments [5].

Aminoglycosides are broad-spectrum antibiotics that inhibit bacterial protein synthesis by binding to the 30S ribosomal subunit and inducing translation errors. Resistance to these compounds commonly arises through enzymatic modification of the antimicrobials, target modification, or increased efflux [6,7]. Recently, streptomycin resistance was related to point and frameshift mutations in chromosomal targets of *Deinococcus geothermalis* [8]; however, acquired aminoglycoside resistance in radiation-resistant *Deinococcus* species has not yet been investigated. Therefore, this study investigated the genomic context of acquired aminoglycoside resistance genes in *D. radiodurans* and evaluated the potential misinterpretation of these traits in publicly available genomes.

## 2. Materials and Methods

### 2.1. Genome Collection

All publicity available *D. radiodurans* genomes, including type strain, substrains, and isolates, were downloaded from the National Center for Biotechnology Information database on 17 February 2026. The genomes included ATCC 13939^T^ (BioSamples: SAMN11313123 and SAMN31235723), ATCC 13939^T^ substr. R1 (BioSample: SAMN11332827), ATCC 13939^T^ substr. R2 (BioSample: SAMN11332829), ATCC 13939^T^ substr. R6 (BioSample: SAMN11332832), ATCC 13939^T^ substr. S1 (BioSample: SAMN11332809), and ATCC 13939^T^ substr. S2 (BioSample: SAMN11332820), CIP104750^T^ (BioSample: SAMEA117660329), DSM 20539^T^ (BioSample: SAMN02743363), R1 (BioSamples: SAMN02603976, SAMN04562548, and SAMN17058454), R1 dM1 (BioSample: SAMN09743852), DRR11 (BioSample: SAMEA9996585), #56 (BioSample: SAMN43044303), BND-54 (BioSample: SAMN14371617), BNK-50 (BioSample: SAMN14371193), BRD125 (BioSample: SAMN26982794), and UBA8872 (BioSample: SAMN08019568). Corresponding metadata and nucleotide sequences were retrieved for all BioSamples.

### 2.2. Bioinformatic Analysis

Genome quality was assessed using CheckM [9]. ResFinder v.4.7.2 [Select provided species: other; Select input type: FASTA (assembled genome/contigs); Threshold for %ID 80; and minimum length 60] from the Center for Genomic Epidemiology [10] and Resistance Gene Identifier v.6.0.5 (Select data type: DNA sequence; Select criteria: perfect and strict hits only; Nudge ≥ 95% identity loose hits to strict: exclude nudge; Sequence quality: high quality/coverage) from the Comprehensive Antibiotic Resistance Database [11] were used to identify acquired antimicrobial resistance genes. Genetic contexts were analyzed using Geneious Prime^®^ v.2026.0.2 and Basic Local Alignment Search Tool (https://blast.ncbi.nlm.nih.gov/Blast.cgi, accessed on 17 April 2026). The sequence comparisons were carried out using Easyfig v.2.2.2 [12].

## 3. Results

**Figure 1:**
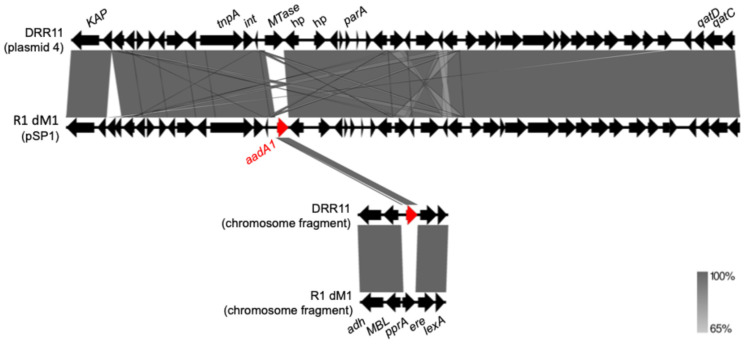
Alignment of plasmid sequences from DRR11 (GenBank accession number: NZ_OV024760) and R1 dM1 (GenBank accession number: NZ_CP031503), and chromosome fragments from DRR11 (GenBank accession number: NZ_OV024759) and R1 dM1 (GenBank accession number: NZ_CP031501). Hypothetical protein, hp. Antimicrobial resistance genes are highlighted in red, whereas other genes are shown in black. Arrows indicate gene orientation, and shaded regions represent sequence similarity between compared regions.

Genome quality assessment indicated high completeness (93.5% to 98.98%) and low contamination levels (<1.2%) across most genomes, supporting the reliability of subsequent analyses. The assembly BRD125 (73.92% completeness) and UBA8872, whose genomic length was too small, were excluded from subsequent analyses. Accordingly, genomes R1 dM1 and DRR11 were identified as carrying the *aadA1* gene. Notably, the *aadA1*-positive sequence of R1 dM1 corresponded to the plasmid pSP1 (~46 kb), a well-characterized shuttle vector used for genetic manipulation in *D. radiodurans*. In contrast, the *aadA1* gene was identified on a chromosome contig (~412 kb) of DRR11 instead of pSP1. Furthermore, *aadA1* was absent in the chromosomes of other *D. radiodurans* genomes, including R1 dM1 (Figure 1). This finding suggests a possible erroneous insertion into the chromosomal contig during genome assembly, rather than representing a true chromosomal integration event.

**Figure 2:**
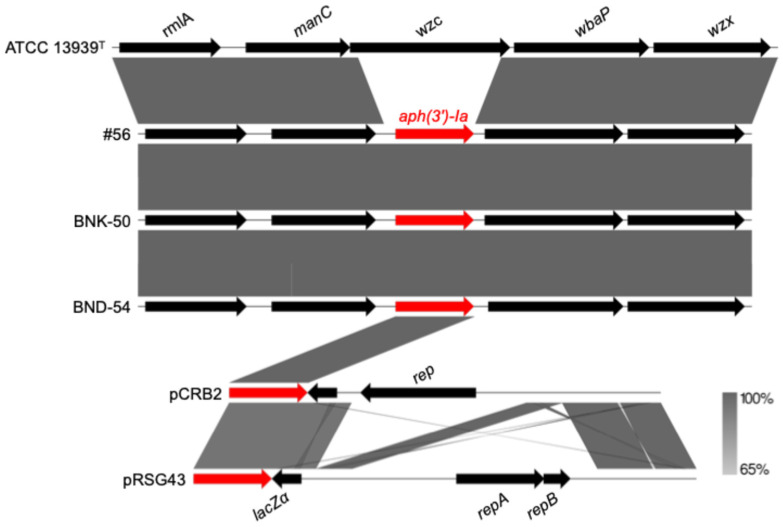
Alignment of chromosome fragments from ATCC 13939^T^ (GenBank accession number: NZ_CP038664), #56 (GenBank accession number: JBHDLK010000002), BNK-50 (GenBank accession number: NZ_CP050117), and BND-54 (GenBank accession number: NZ_CP050121), and plasmid shuttle vectors pCRB2 (GenBank accession number: AB526352) and pRSG43 (GenBank accession number: AB521810). Antimicrobial resistance genes are highlighted in red, whereas other genes are shown in black. Arrows indicate gene orientation, and shaded regions represent sequence similarity between compared regions.

Genomes #56, BNK-50, and BND-54 harbored the *aph(3′)-Ia* gene on chromosome sequences. The surrounding region harboring *aph(3′)-Ia* was conserved across *D. radiodurans* genomes, including the type strain ATCC 13939^T^ (representative genome), in which this gene was absent. This observation indicates that the *locus* itself is part of the native genome and is not inherently associated with AMR. Moreover, the *aph(3′)-Ia* gene is part of shuttle vectors (Figure 2). The presence of *aadA1* and *aph(3′)-Ia* is likely associated with laboratory-derived vectors rather than natural resistome components. Given the high recombination capacity of *D. radiodurans*, these genes may have been integrated into the chromosome following experimental manipulation. Although the conserved chromosomal context and long-read data are consistent with chromosomal integration of *aph(3′)-Ia*, the inconsistent localization of *aadA1* also suggests the possibility of assembly or annotation artifacts.

## 4. Discussion

AMR is recognized as one of the major global health challenges, driven not only by excessive use of antimicrobials but also by the complex interplay between environmental, clinical, and anthropogenic reservoirs [5]. The widespread adoption of whole-genome sequencing has greatly expanded our ability to characterize resistomes across diverse bacterial taxa, including environmental and extremophilic species [13]. This growing set of genomic data has opened new opportunities to explore the diversity and evolution of resistance determinants beyond clinical settings, providing important insights into the ecological dimensions of AMR [13,14].

However, interpretation of AMR in bacterial species with unusual or poorly characterized acquired resistomes has become increasingly challenging because genomic datasets may contain laboratory-derived or exogenous sequences. Recent studies have highlighted that AMR determinants identified in publicly available genomes do not always reflect naturally acquired traits, but may instead originate from experimental manipulation, contamination, or annotation artifacts. This issue has been reported across diverse bacterial taxa, raising concerns about the reliability of resistome analyses when genomic context is not carefully evaluated [15,16,17].

Within the genus *Deinococcus*, current evidence indicates that AMR is primarily driven by intrinsic mechanisms and chromosomal mutations rather than by the acquisition of resistance determinants via mobile genetic elements [1,2]. In *D. geothermalis*, streptomycin resistance has been associated with mutations in ribosome-related genes, including *rsmG*, *rpsL*, and *mthA*, which affect ribosomal structure, function, or RNA modification pathways [8]. These mutations may arise under selective pressures such as oxidative stress and can be further influenced by insertion sequence transposition events, reinforcing the role of genomic adaptability in adaptive resistance [2,18]. Importantly, these findings demonstrate that resistance in radiation-resistant *Deinococcus* species is largely mutation-driven, rather than mediated by horizontally acquired aminoglycoside-modifying enzymes.

In addition, the genomic plasticity of *D. radiodurans* must be carefully considered when interpreting AMR determinants. Even strains considered genetically identical may accumulate significant genomic differences over time, including rearrangements, insertions, and deletions, as a consequence of long-term laboratory propagation and storage conditions. Repeated cycles of growth and preservation, particularly under suboptimal storage conditions, can promote spontaneous genomic changes, while even stationary-phase maintenance may contribute to ongoing genetic variation [19]. These findings indicate that the genome of *D. radiodurans* remains dynamic even under non-stressful laboratory conditions. Consequently, detected genetic differences, including putative resistance genes, may result from laboratory-driven processes such as genetic manipulation and vector use rather than natural evolutionary events. Consequently, caution is required when inferring AMR traits from genomes, particularly for model organisms with extensive experimental histories. Although the genomic context strongly supports our interpretation, reanalysis of raw sequencing reads, independent genome reassembly, and read-mapping-based confirmation would provide additional support and further strengthen the distinction between genuine chromosomal integration, assembly-related artefacts, and laboratory-derived insertions.

In summary, the detection of acquired aminoglycoside resistance genes in *D. radiodurans* genomes should be interpreted with caution. The presence of genes such as *aadA1* and *aph(3′)-Ia*, which are commonly associated with laboratory vectors, is consistent with a laboratory-derived origin, although alternative explanations, including contamination and assembly artefacts, cannot be excluded on the basis of assembled genomes alone. These findings highlight the importance of evaluating genomic context to avoid misinterpretation of AMR in bacterial species, particularly in extremophiles and widely used model organisms. These observations also have broader implications for resistome studies based solely on in silico approaches, emphasizing that AMR determinants should not be interpreted independently of their genomic context, especially in organisms with extensive histories of laboratory manipulation.

## Data Availability

The original contributions presented in this study are included in the article. Further inquiries can be directed to the corresponding authors.
